# Analyzing the persuasion mechanism of AI-generated rumors via the elaboration likelihood model

**DOI:** 10.3389/fpsyg.2025.1679853

**Published:** 2025-11-28

**Authors:** Zhengdong Hou

**Affiliations:** Shantou University, Guangdong, Shantou, China

**Keywords:** generative AI, disinformation, elaboration likelihood model, online rumors, digital literacy

## Abstract

**Background:**

While the technological advancements of Generative Artificial Intelligence are widely recognized, how they reshape the psychological mechanisms of human persuasion and information processing remains underexplored. This study addresses this gap by examining the persuasion mechanisms of AI-generated rumors on internet users, drawing on the Elaboration Likelihood Model (ELM).

**Methods:**

A systematic content analysis was conducted on a large dataset of 11,942 online comments responding to various AI-generated rumors. Using an established coding scheme and a reliability testing procedure, each comment was classified as indicative of either central or peripheral route processing.

**Results:**

The analysis reveals that 90.5% of the comments demonstrated peripheral route processing, with emotional expression as the primary indicator. Only 9.5% of the comments reflected central route processing, most of which involved users providing reasons or evidence, or questioning the source.

**Discussion:**

We argue that the “technological realism” of AI-generated content plays a key role in this pattern. It diminishes users’ ability and motivation to engage in deeper cognitive elaboration, leading them to rely predominantly on the peripheral route for persuasion. These findings extend the Elaboration Likelihood Model to the age of AI and offer practical insights for online platform management, cybersecurity enhancement, and public education in digital literacy.

## 1 Introduction

The profound transformation driven by Generative Artificial Intelligence (GenAI) technology is reshaping the information ecosystem in unprecedented ways (Agrawal et al., 2023; Huang et al., 2024; Kido and Takadama, 2024). While the technical capabilities of these tools, such as Large Language Models (LLMs) and Generative Adversarial Networks (GANs), have received extensive attention (Omeje et al., 2024; Wessel et al., 2025), a more fundamental psychological question remains underexplored: How does this technology alter the core mechanisms of human persuasion and information processing? The misuse of these powerful tools to create and disseminate hyper-realistic disinformation has sparked a potential “infocalypse,” posing an unprecedented threat to social stability (Zhang et al., 2024). However, beyond the technological threat, there lies a critical psychological challenge. When the age-old principle of “seeing is believing” is fundamentally challenged, how do users’ cognitive patterns change? This study aims to address this critical gap.

The core issue is that AI tools enable anyone to easily create fake images and news that are indistinguishable from real information (Zhang et al., 2024). This capability has been exploited by malicious actors, causing tangible social harm (Ghiurău and Popescu, 2024). AI-generated rumors often leverage hyper-realistic visual or textual content centered on themes such as public safety (e.g., fabricated disaster scenes), social conflict (e.g., fictitious group confrontations), or cultural symbols (e.g., altered historical landmarks) (Boutadjine et al., 2025). By tapping into public fear, anger, or identity, these rumors aim to achieve rapid dissemination and public confusion (Bordia and DiFonzo, 2017). More importantly, this behavior has evolved into a new type of cybersecurity threat (Imam, 2025). It is no longer a technical attack in the traditional sense (such as viruses or Trojans), but a form of “social engineering attack” targeting human cognitive vulnerabilities (Burda et al., 2024). Attackers use AI technology as a weapon to mass-produce highly deceptive content at an extremely low cost (Menczer et al., 2023). Their goal is to manipulate public opinion, undermine social trust, and even affect the stable operation of critical infrastructure (Schreiber et al., 2021).

Although the phenomenon of AI-generated rumors has received widespread attention, there is still a lack of in-depth empirical research on the psychological mechanisms that persuade users (Paschen, 2020). The old saying, “seeing is believing,” is now fundamentally challenged. How do users then change their ways of processing information? To answer this question systematically, our study uses a classic theory from communication and social psychology: the Elaboration Likelihood Model (ELM). The ELM provides a powerful analytical framework for understanding how individuals process persuasive information (Petty et al., 2015). This model distinguishes between two routes of persuasion: the central route, which involves careful and effortful consideration of message arguments, and the peripheral route, which relies on superficial cues for quicker judgment. This distinction aligns closely with dual-process theories of human cognition, notably Kahneman and Tversky’s influential “two-system theory.” This theory posits that human thinking operates through System 1 (fast, intuitive, emotional, and effortless processing) and System 2 (slow, deliberative, logical, and effortful processing) (Kahneman, 2011). The peripheral route in ELM largely mirrors System 1 processing, while the central route corresponds to System 2. Understanding this underlying cognitive architecture is crucial, as AI-generated rumors are often designed to bypass System 2 thinking and directly engage System 1 through emotional and heuristic appeals.

Based on this, the core objective of this study is to investigate: How do AI-generated rumors persuade online users? Specifically, this study aims to identify whether users are more inclined to follow the central route for logical reasoning or are more easily influenced by peripheral cues when faced with AI rumors. The findings of this study will provide evidence-based strategic recommendations for platform governance, policy-making, and public education.

## 2 Materials and methods

### 2.1 Theoretical framework

**Figure 1:**
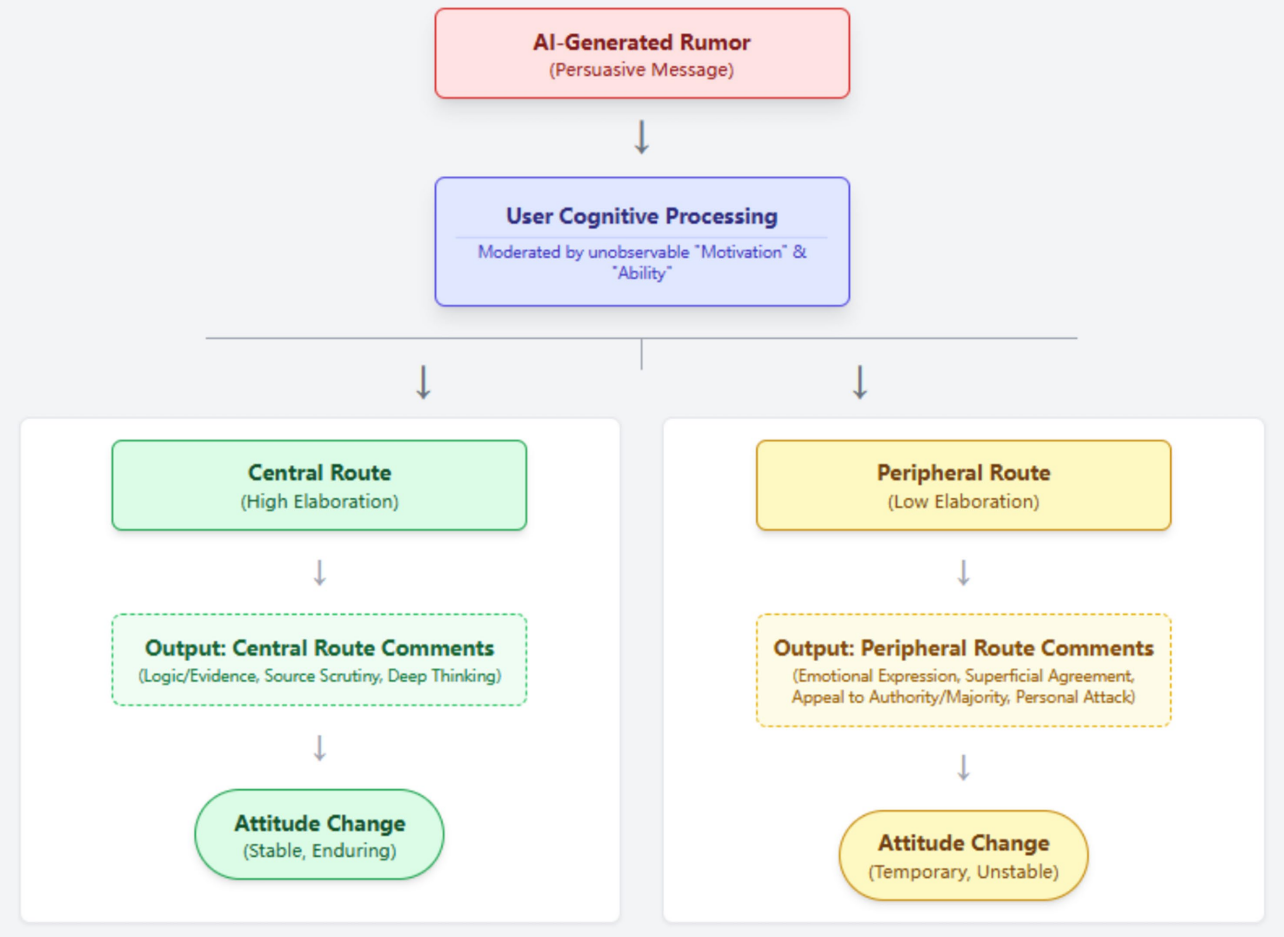
Research protocol.

This study employs content analysis, guided by the ELM as its theoretical framework (Petty and Cacioppo, 1986). The ELM theory posits that individuals process persuasive messages via two distinct cognitive routes: the central route or the peripheral route (SanJosé-Cabezudo et al., 2009). As the data for this study are sourced from public online comments, the focus is on analyzing user comments as the “observable output” of the cognitive process. By examining the characteristics displayed in the comments, we can identify the cognitive route taken by the user. As illustrated in Figure 1, after a user is exposed to an AI rumor, if their comment shows deep thinking and logical analysis, it can be considered an indicator of central route processing (Li and See-To, 2024; Choi and Stvilia, 2015; Srivastava and Saini, 2022). Conversely, if it shows reliance on superficial cues like emotions or source credibility, it is considered an indicator of peripheral route processing (Dedeoğlu et al., 2021; Srivastava and Saini, 2022). Through the quantitative analysis of these observable indicators, this study aims to reveal the macroscopic picture of the persuasion process of AI-generated rumors.

### 2.2 Data collection and processing

Our study systematically collected human-generated user comments from several mainstream Chinese online platforms between July 14 and 16, 2024, to build a representative dataset. The data sources included popular short-video and social media platforms such as Douyin and Bilibili, as well as the comment sections of major online news portals.

Importantly, all comments in our dataset were written by human users in response to AI-generated rumor cases, rather than AI-generated comments themselves. The rumors examined in this study included several widely circulated cases, such as the “Hanging Monastery Collapse,” “Dali Traffic Accidents,” and “Mount Emei Man-Monkey War.”

An initial dataset of 12,795 comments was collected. During the subsequent data cleaning phase (July 17–24), a total of 853 invalid entries were removed, including advertisements, duplicate content, empty lines, and comments consisting only of emojis or “@” mentions. The final dataset consisted of 11,942 valid, human-generated comments.

For example, a typical comment in the dataset might read: “This video looks fake — the shadows do not match; it must be AI-generated.” Such comments reflect real users’ reactions to AI-generated rumor content and help capture the public’s perception and reasoning regarding this phenomenon.

### 2.3 Development of the coding scheme

Based on the theoretical framework of the Elaboration Likelihood Model (ELM), this study established a detailed coding scheme to systematically classify the cognitive processing routes reflected in user comments. The core concept of this framework is “Cognitive Elaboration,” which refers to the extent to which an individual engages in careful and effortful thinking about the arguments contained in a persuasive message (Barone and Smith Hutchings, 1993; Bloemer et al., 2009). High cognitive elaboration means a person has put a lot of mental effort into carefully and logically analyzing information. On the other hand, low cognitive elaboration means they rely on mental shortcuts. This leads to quicker, but less thorough, judgments (O’Keefe, 2013; Chaiken, 1980; Stephenson et al., 2001; Wagner and Petty, 2022). Accordingly, the coding scheme is structured around two primary dimensions: the “Central Route” and the “Peripheral Route,” which represent high and low levels of cognitive elaboration, respectively (SanJosé-Cabezudo et al., 2009; El Hedhli and Zourrig, 2023). Each primary dimension is further divided into several secondary indicators to capture specific comment characteristics (Table 1).

**Table 1:** Coding scheme for content analysis of user comments based on the ELM.

Primary dimension	Secondary dimension	Definition
Central route	Logic/evidence	The comment supports or refutes the rumor by providing factual evidence, logical reasoning, or pointing out internal contradictions.
	Source questioning	The comment explicitly questions the credibility, motives, or identity of the information source.
	Deep thinking	The comment goes beyond the event itself to discuss deeper societal issues, technological ethics, or systemic causes.
Peripheral route	Emotional expression	The comment primarily expresses strong personal emotions such as fear, anger, surprise, or sadness, without substantive argumentation.
	Superficial agreement	The comment simply expresses agreement or support, or indicates a forwarding action, without providing additional arguments or analysis.
	Appeal to authority/majority	The comment forms a judgment by citing authoritative sources or the opinions of the majority.
	Personal attack	The comment does not address the content itself but attacks the character, intelligence, etc., of the publisher or other commenters.

The Central Route, indicative of high cognitive elaboration, is activated when an individual is both motivated and able to process the core arguments of a message critically (Bhattacherjee and Sanford, 2006; Petty and Cacioppo, 1984). This route was operationalized through three secondary indicators (Angst and Agarwal, 2004). The first, Logic/Evidence, captures comments that attempt to verify the veracity of the information by providing counter-evidence, identifying internal logical inconsistencies, or applying do-main-specific knowledge, thus reflecting a focus on the quality of the message’s content (Ikuenobe, 2001; Zhang et al., 2015; Montgomery and Gray, 2017). Source Questioning shows a more advanced kind of critical thinking. Here, people look beyond the message itself and focus on where it came from. We mark comments under this category if they question the publisher’s trustworthiness, who they are, or why they shared the information (Pilgrim et al., 2019; Slater and Rouner, 1996). The highest level of deep thinking is called Deep Thinking. In these comments, people’s thoughts go beyond just the specific event. They discuss bigger issues like how society is affected, ethical rules for technology, or ideas for how to fix problems in the system (LeBoeuf and Shafir, 2003; Simon, 1979).

The Peripheral Route shows a low level of deep thinking. People use this route when they do not want to or cannot fully understand the main points of a message. Instead, they depend on simple “peripheral cues” to quickly make a decision (Wegener et al., 2010; Wegener et al., 1994). We measured this route using four types of indicators. The most common one is “Emotional Expression.” In this case, the commenter’s feelings are mostly shaped by emotions from the message itself. They do not rely on logical thinking. Instead, their comments mainly show their feelings without giving clear reasons (Servi and Elson, 2015; Kleef et al., 2015; Trapani and Murdaugh, 2022). Another common mental shortcut is called “Appeal to Authority/Majority.” In this shortcut, people let others make their judgment for them. They decide to believe or not believe something based on what an authority figure says. An example is comments like “Waiting for the official announcement.” Or, they might believe something because most people seem to agree (Brownlee, 2020; Knyazev and Oosterhuis, 2022). Additionally, Personal Attack involves comments that completely bypass the message content and instead direct ad hominem attacks toward the personal character, intelligence, or motives of the publisher or other commenters, representing an irrational response based purely on stance and emotion (Arnold, 1994; Garssen, 2009; Battaly, 2010). Finally, Superficial Agreement represents the lowest level of cognitive engagement, characterized by simple expressions of agreement or support, or actions like liking, forwarding, or using emojis, which indicate a passive reception of the information without additional thought (Choi et al., 2023).

### 2.4 Coding procedure and reliability test

To ensure the objectivity and systematicity of the analysis, a manual annotation (coding) procedure was employed, conducted by two trained human coders. Each coder independently labeled every comment according to a predefined coding scheme derived from the Elaboration Likelihood Model (ELM). Specifically, each comment was classified as reflecting either the central route (analytical and evidence-based reasoning) or the peripheral route (emotion-driven or heuristic responses).

Prior to the formal coding, both coders underwent training using a subset of sample comments to ensure a shared understanding of the category definitions and to establish consistent annotation standards. After training, all 11,942 valid comments were manually coded. To test the reliability and stability of this process, a 20% random subsample (*n* = 2,388) was independently re-coded by the same coders two weeks after the initial round. This proportion is widely accepted in content analysis as a suitable subset for evaluating intercoder reliability while maintaining methodological rigor and feasibility (Lombard et al., 2002; Burla et al., 2008; Geiß, 2021).

Inter-annotator agreement (IAA) was calculated using Cohen’s Kappa, resulting in a value of 0.89, which indicates “almost perfect” agreement (McHugh, 2012). This result demonstrates the high reliability of the manual annotation process. In cases of disagreement, the coders discussed and resolved the differences through consensus to ensure the overall accuracy of the dataset classification.

For illustration, comments such as “This video must be fake; the lighting and shadows are inconsistent” were coded as central-route responses, while comments like “AI is scary these days—you cannot believe anything online” were coded as peripheral-route responses.

## 3 Results

### 3.1 Descriptive statistics: the dominance of the peripheral route

Overall classification statistics for all comments clearly show the overwhelming dominance of the peripheral route in user responses (Table 2). Of all 11,942 comments, 10,807 were classified as peripheral route, accounting for a high of 90.5%, while only 1,135 comments reflected central route processing, accounting for 9.5%.

**Table 2:** Overall distribution of information processing routes.

Processing route	Number of comments	Percentage
Central route	1,135	9.5
Peripheral route	10,807	90.5
Total	11,942	100

### 3.2 Thematic influence on user response patterns

This study’s dataset is designed to capture general reactions to AI-related rumors. However, when comments are grouped by different themes, clear differences emerge. The topic of each rumor plays a key role in shaping both the focus and the emotional intensity of public responses (see Table 3).

**Table 3:** Comparison of dominant comment indicators across different rumor themes.

Rumor theme	Total comments	Dominant central route indicator	Dominant peripheral route indicator
Public safety/disaster	3,821	Common sense	Emotional expression (fear/shock)
Social conflict/morality	4,538	Fact-checking	Emotional expression (anger/disgust)
Cultural heritage/identity	3,583	Historical knowledge	Personal attack/emotional expression (anger)

The analysis shows that different themes trigger distinct cognitive and emotional reactions. For Public Safety/Disaster rumors (N = 3,821), which directly threaten personal security, the dominant peripheral response is Fear/Shock, while rational rebuttals often rely on accessible Common Sense. For Social Conflict/Morality rumors (N = 4,538), which touch upon social values and justice, the primary emotion is Anger/Disgust, and central route processing is more inclined towards Fact-Checking to establish blame. For Cultural Heritage/Identity rumors (N = 3,583), which can be perceived as an attack on collective identity, the response is also dominated by Anger, but is uniquely characterized by a higher proportion of Personal Attacks against the perceived perpetrators.

### 3.3 Analysis of central route processing: a minority’s rational resistance

Among the 1,135 central route comments, “Logic/Evidence” was the most common indicator, accounting for 50.3% (571 comments). This category includes various forms of rational engagement. The most direct form was rebuttal through personal experience, as seen in comments like, “I just went to the Hanging Monastery this morning, scared me to death, AI fakes are terrifying.” Another common form was using common sense and logical deduction to identify flaws, for example, “The Hanging Monastery has been hanging for over 1,000 years, how could it suddenly collapse?” and “It collapsed but the top is still so intact, obviously AI.” Some users also engaged in basic technical analysis by pointing out inconsistencies in the media itself, such as identifying that the monkeys in the “Mount Emei Man-Monkey War” video were not the correct species: “The monkeys being shot in the video are long-tailed monkeys from Africa, not the short-tailed macaques of Mount Emei.”

“Source Questioning” was the second most frequent indicator, at 29.8% (338 comments). These comments shifted focus from the content to the creator, primarily through motive attribution. Users often pointed to the pursuit of online traffic as the core motive, with remarks like, “Who profits from this? Whoever makes a fortune is at fault,” and “People on the internet can be so immoral just for traffic.”

“Deep Thinking” was the least frequent but most profound indicator, at 19.9% (226 comments). These comments moved beyond the specific event to discuss systemic issues. This included proposing governance solutions, such as, “The penalty for rumor-mongering is too light,” and “AI-generated videos should have a special label.” It also involved analyzing societal impacts, for instance, “The creation of malicious rumors … exposes the chaos of a traffic-first internet … Rumor-mongers must pay the legal price to deter others!” Furthermore, some comments reflected on media literacy, noting differences in discernment abilities across demographics: “Young people like us can basically tell the difference, but those middle-aged and elderly people really just forward whatever they see.”

### 3.4 Analysis of peripheral route processing: the anatomy of emotional contagion and cognitive shortcuts

Among the 10,807 peripheral route comments, “Emotional Expression” was the absolute dominant indicator, accounting for 75.1% (8,116 comments). This category primarily involved expressions of anger and condemnation, often calling for severe punishment for the rumor-mongers. Comments like “Strictly investigate and punish this type of garbage!!!!” and “Should be severely punished, otherwise fake news for traffic will be everywhere” were widespread. Another significant emotion was fear and anxiety, particularly in response to rumors about cultural heritage destruction, as seen in “Scared me to death, AI fakes are terrifying.” A third type was declarative statements of stance, such as “I hate the monkeys of Mount Emei,” which, although related to the topic, lacked logical argumentation and were purely emotional judgments.

“Appeal to Authority/Majority” was the second most important indicator, at 10.0% (1,081 comments). This cognitive shortcut manifested in two ways. The first was a reliance on official power, where users offloaded the responsibility of judgment to authorities, with comments like “Have the police caught the rumor-monger? If not, it’s not a rumor.” The second was following the crowd, where users’ opinions were swayed by the perceived majority view, as in “So many people are saying it, it must be true.”

“Personal Attack” accounted for 9.8% (1,059 comments). These comments entirely bypassed the content of the rumor and instead launched ad hominem attacks on the creators or other users. Examples include moral judgments like “This person is not afraid of retribution” and direct insults.

“Superficial Agreement” was the least frequent indicator at 5.1% (551 comments), representing the lowest level of cognitive engagement. This included simple expressions like “support,” “agree,” “forwarded,” the use of emojis, or simply tagging other users to draw their attention, indicating a passive reception and spread of information.

## 4 Discussion

A systematic analysis of the empirical data, derived from a substantial corpus of online commentary, indicates that in the context of AI-generated rumors, user response patterns exhibit a significant predisposition toward the peripheral route of information processing. This primary finding, wherein 90.5% of responses align with peripheral processing, not only substantiates the applicability of the Elaboration Likelihood Model (ELM) within a novel technological milieu but, more critically, it furnishes a robust empirical foundation for a thorough examination of the potential systemic impacts exerted upon the ELM’s principal moderating variables—namely, “motivation” and “ability”—within the contemporary era of artificial intelligence. The thematic analysis further enriches this understanding by revealing that the subject matter of the rumor acts as a significant moderating variable, shaping the specific nature of these cognitive and emotional responses.

### 4.1 Deeper analysis: the dangerous convergence of technological realism and cognitive laziness

The pronounced prevalence of the peripheral route (90.5%) constitutes the study’s most consequential and exigent finding. This statistical asymmetry is indicative not merely of a numerical disparity but signifies a qualitative manifestation of a systemic vulnerability within the contemporary information architecture. The phenomenon elucidates a precarious confluence of two potent factors: the perceived realism and its persuasive power inherent in artificial intelligence’s technological realism and a pervasive public inclination toward cognitive indolence. This nexus presents a formidable threat to cybersecurity and societal stability, effectively transmuting artificially generated rumors from rudimentary disinformation into sophisticated instrumentalities for cognitive warfare.

An examination of the data indicates that artificially generated rumors function as uniquely potent mechanisms for the large-scale fomentation of emotional polarization and the execution of social engineering. The observation that approximately three-quarters of comments processed via the peripheral route consist of purely emotional articulations substantiates the proposition that such rumors operate as content designed to provoke strong emotions. Their operational design appears to circumvent deliberative ratiocination in favor of directly eliciting visceral, affective responses, thereby inflaming latent societal tensions and anxieties. Such mass affective mobilization is subject to weaponization for the purposes of creating acute social cleavages, inciting animosity, and corroding the foundations of social trust, which are cardinal objectives within the strategic calculus of cognitive domain attacks in contemporary cyber warfare. The minimal expenditure and maximal efficiency associated with the generation of such content via artificial intelligence empower malevolent entities to orchestrate sustained, large-scale affective assaults upon the public sensorium, representing a threat vector heretofore largely unattainable.

Furthermore, the conspicuously low incidence of central route processing (9.5%) denotes a pervasive degradation of critical ratiocinative faculties, a condition that may be conceptualized as a critical human-centric vulnerability within the cybersecurity paradigm. Artificially generated rumors are observed to exploit this cognitive deficiency with unparalleled scale and scope. The verisimilitude of the content serves to diminish public vigilance, thereby reducing the motivational impetus required to undertake the cognitively strenuous tasks of factual verification and logical scrutiny. Such conditions cultivate a fertile environment for expansive social engineering campaigns, wherein the populace inadvertently functions as a propagation vector for disinformation. This situation can also lead to what we call the “liar’s dividend.” This means people are constantly seeing very realistic fake content. Because of this, they start to doubt if real information is true. In the end, this causes a general loss of trust in all sources of information, including well-known news media and government agencies. This widespread doubt in society gives a big advantage to groups that want to cause social harm. This is because it stops people from having helpful public discussions and making good decisions together.

Lastly, the observed dependence on authoritative cues (10.0% of peripheral comments) is indicative of a disquieting trajectory toward the externalization of critical judgment and a consequent state of cognitive atrophy. Although deference to authoritative bodies is a requisite component of societal function, an excessive reliance thereupon establishes a critical single point of failure. In circumstances where official channels of communication exhibit latency in their response—a frequent eventuality given the celerity of AI-generated content propagation—or should their credibility be compromised, an informational vacuum emerges, which is readily exploitable by malevolent actors. This posture of passivity signifies an abdication of individual epistemic responsibility concerning information verification, rendering the public exceptionally vulnerable to manipulation. Protracted reliance on such external validation mechanisms may precipitate a condition of “cognitive atrophy,” wherein the faculties for independent critical thought deteriorate from a state of prolonged disuse. Fundamentally, for a substantial majority of users, the veracity of the information is relegated to a position of secondary importance, subordinate to the pursuit of immediate affective gratification or the facility of adhering to simplistic peripheral cues. This confluence of factors engenders a precarious environment, ripe for the manifestation of cognitive security threats capable of inducing societal destabilization from within.

### 4.2 The attenuation of cognitive elaboration via technological verisimilitude

The Elaboration Likelihood Model (ELM) suggests a key idea. For people to use the central route to process information, they need both enough motivation and ability. These two things must be present at the same time in the person receiving the message. The empirical results of this study, however, reveal a stark deviation from this deliberative ideal, with 90.5% of user responses defaulting to the peripheral route. This pronounced and consistent asymmetry across diverse rumor topics strongly suggests the operation of a universal influencing variable that systematically suppresses these two essential preconditions for cognitive elaboration. A theoretical proposition is advanced herein that this variable is the intrinsic technological characteristic of the medium itself—specifically, the hyper-realistic verisimilitude of AI-generated content. The causal pathway for this assertion is twofold, directly and simultaneously impacting the core moderators of the ELM.

Firstly, with respect to processing ability, the hyper-realism of AI-generated content functions to dismantle the cognitive safeguards that individuals typically employ to detect falsity. Conventional forms of disinformation frequently exhibit discernible logical or factual incongruities—anomalies that function as cognitive tripwires, alerting the recipient to the potential fallaciousness of the information and thereby triggering critical scrutiny. In contrast, AI-generated content substantially mitigates such rudimentary errors, presenting a degree of visual and textual fluency that is often indistinguishable from veridical information. Consequently, even should a user possess the inclination to critically evaluate the content, they may be deficient in the requisite technical expertise and analytical tools to deconstruct such sophisticated fabrications. This technological barrier effectively causes a diminution of their “ability” to engage in central route processing.

Secondly, in relation to processing motivation, the realism of AI-generated content may also precipitate a profound erosion of the impetus for cognitive elaboration. The cognitive heuristic of “seeing is believing” is a deeply entrenched and efficient, albeit fallible, mechanism for assessing reality. When an AI-generated rumor is presented in a format of high fidelity that perfectly mimics authentic media, its superficially credible presentation is likely to lower the threshold of user skepticism. Insofar as the information appears veridical, the perceived exigency of expending finite cognitive resources on in-depth verification—that is to say, the motivation for elaboration—is consequently and significantly diminished. The content’s realism provides a compelling peripheral cue that discourages the allocation of mental effort required for central processing.

Based on the foregoing analysis, a theoretical supplement is proposed: in the milieu of artificial intelligence, “Technological Realism” may be conceptualized as a pivotal antecedent variable that modulates the likelihood of elaboration. It is posited that a direct inverse correlation exists between the degree of technological realism and a user’s ability and motivation to engage in central route processing, which in turn leads to the peripheral route becoming the predominant conduit of persuasion. This construct offers a more profound explanatory framework for the findings of this study. We saw strong feelings like fear or anger in the public. This happens largely because of “technological realism.” This concept allows fake stories to look so real that they directly cause strong, basic emotional reactions. In this way, people often bypass thinking things through logically.

### 4.3 Implications for cybersecurity and governance

The findings of this inquiry possess not only theoretical import but also direct prescriptive value for the domains of cybersecurity practice and public policy formulation. AI generated rumors mainly work by using the peripheral route. They especially depend on emotions and cues from authorities. Therefore, our defense strategies must do more than simply check facts. It is imperative to construct a multi-layered defensive framework that synthesizes both social and technical methodologies.

#### 4.3.1 From content moderation to cognitive assistance

Data from this study show that some user comments explicitly suggest that “AI should be cancelled, it’s too fake.” This directly reflects the public’s demand for technological transparency and control. Platform operators and regulators should promote the establishment of mandatory labeling or digital watermarking standards for AI-Generated Content (AGC). This is not for censoring content, but to provide users with a crucial “peripheral cue”: “AI generated this content.” This label can trigger alertness, reducing the cognitive desensitization caused by “technological realism” and creating conditions for initiating central route processing.

#### 4.3.2 Treating AI rumors as analyzable attack campaigns

This study observed a typical AI rumor attack pattern: an attacker uses real materials, exaggerates them with AI, and distributes them on multiple platforms for traffic. Cybersecurity analysts can build threat models based on this pattern (TTPs—Tactics, Techniques, and Procedures). Furthermore, AI-generated content may have “linguistic fingerprints.” Future research could leverage Natural Language Processing (NLP) and neural networks to train detection models specifically for identifying this “AI writing style,” shifting from passive debunking to proactive “threat hunting.”

#### 4.3.3 Reshaping critical thinking for the digital age

Since the peripheral route is dominant, enhancing public digital literacy education is particularly urgent. Citizens need to understand that when they see a piece of information and feel intense anger or fear, this is itself a warning sign that they may be in a peripheral route processing mode. Education should guide the public to form a habit: the more emotional the reaction, the more one should pause before sharing and actively seek cross-verification. Data show that 10.0% of peripheral route comments rely on “appeal to authority/majority,” which indicates that official debunking is still effective. Therefore, establishing fast, authoritative, and easily accessible debunking channels is crucial.

## 5 Conclusion

This study, using the ELM as a theoretical lens, delved into the persuasion mechanisms of disinformation in the AI era. The core finding is that peripheral route processing (accounting for 90.5%) is the dominant mode for users responding to AI rumors. The study’s primary theoretical contribution is the proposition of “technological realism” as a key antecedent variable that systematically undermines users’ ability and motivation for central route processing, thus offering a critical update to the ELM framework for the digital age. This concept explains how the hyper-realism of AI-generated content short-circuits rational deliberation in favor of heuristic cues like emotion and source appearance. The study further reveals that the thematic content of a rumor acts as a significant secondary variable that shapes the specific nature of these public responses. The contribution of this study also extends to cybersecurity, defining AI-generated rumors as a cognitive domain attack and providing a new perspective for cyber threat intelligence. The proposed multi-layered defense framework offers concrete strategies for managers, security specialists, and policymakers.

## 6 Limitations and future research

While this study provides valuable insights into the persuasion mechanisms of AI-generated rumors, it is important to acknowledge its limitations, which in turn suggest crucial avenues for future research.

First, the data were collected exclusively from Chinese social media platforms, such as Douyin and Bilibili, during a specific period. While this provides a deep understanding of the phenomenon within this particular cultural context, it also means that the observed patterns of information processing and peripheral reliance, particularly on emotional and authoritative cues, might be uniquely shaped by Chinese societal norms, information consumption habits, and existing trust dynamics within its digital ecosystem. For instance, the emphasis on collective consensus or deference to official statements might differ significantly in Western contexts, where individualistic approaches to information validation might prevail. Therefore, the generalizability of these findings to other cultural settings or regions with varying levels of digital literacy remains to be fully explored. Future research could conduct cross-cultural comparative studies to explore whether persuasion mechanisms and the prominence of “technological realism” differ across platforms and cultures.

Second, our sample consists solely of online users who actively chose to post comments. This group may not be fully representative of the entire online population, as those who comment online are often the most emotionally engaged or opinionated, and therefore potentially more prone to peripheral route processing than passive information consumers (a potential “self-selection bias”). This inherent bias in our dataset might have inflated the observed dominance of peripheral responses, especially Emotional Expression and Appeal to Authority/Majority, thereby potentially overstating their prevalence compared to a broader user base. Future studies could employ survey or experimental methods to capture responses from a more diverse and representative sample, allowing for a more balanced assessment of these processing routes.

Third, the content analysis method allows for inferences about cognitive processing routes based on textual output, but it cannot directly measure the underlying psychological processes. Although our coding scheme is robust, the interpretation of a comment as reflecting a specific processing route is still an inferential step. Future research should complement this approach with experimental designs. For example, researchers could expose participants to controlled AI-generated stimuli and directly measure attitude changes, cognitive load, and emotional responses to establish clearer causal links, particularly concerning how “technological realism” manipulates these cognitive and affective pathways.

Finally, regarding the conceptualization of “technological realism,” while it serves as a crucial interpretive concept in our current framework, we acknowledge the need to sharpen its operationalization for future empirical work. We propose that future studies could explore ways to measure or manipulate this concept more concretely. For instance, “technological realism” could be experimentally manipulated by varying the fidelity (e.g., visual quality, contextual coherence, emotional appropriateness) of AI-generated content in controlled settings.

Alternatively, its perception could be assessed through self-report measures of authenticity or believability among participants. Offering these possibilities would make “technological realism” more than a descriptive label, giving it a clearer place in ongoing empirical research aimed at understanding its precise impact. Building on these methodological advancements, future work could also explore the cognitive profiles of individuals who are more susceptible or resistant to AI-generated rumors. Investigating the role of individual differences, such as digital literacy, cognitive reflection, and personality traits, would provide a more nuanced understanding of this critical issue and allow for tailored interventions that enhance central route processing among vulnerable populations.

## Data Availability

The original contributions presented in the study are included in the article/supplementary material, further inquiries can be directed to the corresponding author.
